# Thinking multi-scale to advance mechanobiology

**DOI:** 10.1038/s42003-020-01197-5

**Published:** 2020-08-21

**Authors:** Marco Fritzsche

**Affiliations:** 1 Kennedy Institute of Rheumatology, University of Oxford, Roosevelt Drive, Oxford, OX3 7FY UK; 2 Rosalind Franklin Institute, Harwell Campus, Didcot, OX11 0FA UK

## Abstract

We are inviting submissions of articles on the role of mechanobiology in health and disease with the aim of publishing high quality research devoted to advance our understanding of mechanics shaping biological function. We are also happy to present a Collection of papers already published in our journal in this exciting field.

M**echanobiology is the elephant in the room: it has long been overlooked, despite the fact that biomechanics is known to influence many functional features of biological life.** Although the biomechanical frontiers that cells face have perhaps always been most evident in the context of the physical behaviour of cells, advances in the past few years have demonstrated the impact of mechanical force on cellular function in many biological systems in health and disease.

New insights in the field of mechanobiology reveal that cell function is directly influenced by both the mechanical properties and forces of the surroundings^1^. In the ever changing physical world, the human body and its tissues primarily rely on their individual cells to process mechanical information, which have evolved strategies to exploit a combination of an active skeleton and specialised protrusions, allowing them to receive and respond to mechanical cues at a range of spatial and temporal frequencies. Crucially, the secret to the success of this strategy is the inseparable relationship between biology and mechanics, often resulting in the emergence of distinct properties depending on the observation frequency.

Beyond the ability of cells to assess the physical world, they also must integrate and interpret mechanical information to shape their function. Through mechanical feedback, living cells can adapt the biomechanics of their own bodies to their immediate microenvironment, intricately serving their physiology. This process of mechanosensation allows biological systems, from single cells to whole tissues, to translate biomechanical cues into cellular physiological changes that potentiate their organisation and orchestration in space and time. Through these spheres of physical, transcriptional, and metabolic influence, mechanical determinants govern the behaviour and biological function of individual cells and multi-cellular ensembles, and even of entire organs. Understanding the sub-cellular mechanisms, the ramifications for cell tissues, and ultimately the biological significance of mechanobiology on the organism as a whole represents a grand challenge across the biological, physical, and biomedical sciences. Very similar, if not identical concepts of cells exploiting mechanobiology have been observed across biomedical disciplines. One of the most exciting findings regarding the mammalian immune system is the realisation that the immune response can be perceived as the action of a single multi-scale super-organism actively processing the influence of its biochemical and biomechanical environment^2^. This astounding machinery results in millions of cells communicating through a combination of chemical and biomechanical signals to organise and orchestrate their behaviour and function against a pathogenic threat. However, diseases can still find a way to avoid the multi-scale machine, underscoring the need for more research in this area. A striking example of circumventing the ability of the immune response to integrate mechanics is the strategy employed by cancerous tumours. Tumour cells can isolate themselves—through mechanical stiffness maturation—from healthy tissues^3^, thereby protecting tumour growth from the deadly function of immune killer cells. Mechanical maturation is a common concept not only in immunopathology but also in health^4^, as for instance apparent in the ageing of many of our organs or during skin and wound healing, vascularisation, lymphatic function, bone, neuron activity, as well as the advanced age-related deterioration of corneal epithelial cells of the eye. What all these examples have in common is the prospect of curing disease by tuning the control of mechanical force over biological function at multiple scales from molecules, to single cells, tissues and organs. Consequently, current research must involve multi-scale approaches, both literally in the context of understanding the hierarchy and interconnectivity of biology and mechanics and more figuratively in terms of involving research efforts from different perspectives. By unravelling the web of complexity inherent to mechanobiology, the potential of this field to contribute to the future directions of basic research and clinical applications is promising.

We at *Communications Biology* invite and welcome such efforts to advance mechanobiology. Over the period of the last two years since our launch in January 2018, we have aimed to provide a home to basic research shaping the emerging interface of where biology meets mechanics. Our Collection published today illustrates how biomechanical factors shape cellular function through influencing the structural integrity, morphology, and dynamics of single cells, ensembles, and tissues. Recent examples are the study by Professor Boon and colleagues identifying a lncRNA involved in shear stress sensing in vasculature^5^ and work by Professor Gramolini’s lab describing a culture system to study the function of cardiomyocytes^6^.

With its broad and interdisciplinary focus, *Communications Biology* is ideally placed to provide a venue for research at the interface of biology and mechanics. We are inviting submissions on mechanobiology in health and disease with the intent of enhancing the visibility of current research investigating the ways in which mechanics shapes biological function. In particular, we are interested in (but not limited to) studies of the immune system, aging, skin, vascularisation, lymphatics, bone, neurons, and the eye. We are also happy to receive studies focusing on mechanical aspects of development. Comments, Perspectives and Reviews will also be considered.

With its broad and interdisciplinary focus, Communications Biology is ideally placed to provide a venue for research at the interface of biology and mechanics.

We encourage our authors to not hold back on their incipient research ideas, and to think multi-scale for inspiration and advancement of the field of mechanobiology. As we at *Communications Biology* embark on further developing this Collection, we would like to think of you as a crucial contributor to making it a success story. As I know well from my own research activities leading the interdisciplinary Biophysical Immunology Laboratory (www.bpi-oxford.com) between the Rosalind Franklin Institute and the Kennedy Institute at the University of Oxford, finding visibility for biological research studies led from the point-of-view of physics can be challenging. We hope that our Collection will inspire and point to future directions in the mechanobiology community.

We encourage our authors to not hold back on their incipient research ideas, to think multi-scale for inspiration and advancement of the field of mechanobiology.
